# Risk of Ischaemic Heart Disease in Patients with Inflammatory Bowel Disease: Cohort Study Using the General Practice Research Database

**DOI:** 10.1371/journal.pone.0139745

**Published:** 2015-10-13

**Authors:** Helen Close, James M. Mason, Douglas W. Wilson, A. Pali S. Hungin, Roger Jones, Greg Rubin

**Affiliations:** 1 School of Medicine, Pharmacy and Health, Durham University, Stockton-on-Tees, United Kingdom; 2 Department of General Practice and Primary Care, King’s College, London, United Kingdom; Laikon Hospital, GREECE

## Abstract

**Objective:**

Patients with inflammatory bowel disease (IBD) demonstrate an inflammatory response which bears some similarities to that seen in ischaemic heart disease (IHD). The nature of the association of IBD with IHD is uncertain. We aimed to define the extent and direction of that association.

**Design:**

This retrospective cohort study examined records from patients aged ≥ 15 years with IBD from 1987–2009 (n = 19163) who were age and gender matched with patients without IBD (n = 75735) using the General Practice Research Database. The primary outcome was the hazard ratio for IHD.

**Results:**

A higher proportion of IBD patients had a recorded diagnosis of IHD ever, 2220 (11.6%) compared with 6504 (8.6%) of controls. However, the majority (4494, 51.5%) developed IHD prior to IBD diagnosis (1404 (63.2%) of IBD cases and 3090 (47.5%) of controls). There was increased IHD incidence in the first year after IBD diagnosis. Mean age at IHD diagnosis was statistically similar across all IBD groups apart from for those with Ulcerative Colitis (UC) who were slightly younger at diagnosis of angina compared to controls (64.5y vs. 67.0y, p = 0.008) and coronary heart disease (65.7y vs.67.9y, p = 0.015). Of those developing IHD following IBD diagnosis, UC patients were at higher risk of IHD (unadjusted HR 1.3 (95% CI 1.1–1.5), p<0.001) or MI (unadjusted HR 1.4 (95% CI 1.1–1.6), p = 0.004).

**Conclusion:**

Although IHD prevalence was higher in IBD patients, most IHD diagnoses predated the diagnosis of IBD. This implies a more complex relationship than previously proposed between the inflammatory responses associated with IHD and IBD, and alternative models should be considered.

## Introduction

The inflammatory bowel diseases (IBD) ulcerative colitis (UC) and Crohn’s Disease (CD) are disorders of uncertain origin. In both UC and CD, an exaggerated immune response to unknown antigens is evident [1] and a number of cytokines with pro-inflammatory effects have been described, notably IFN-ƴ and IL-17/IL-22 in Crohn’s disease, IL-13, TNF-α, IL-1β, IL-6 and TL1A in ulcerative colitis.[2, 3] The inflammatory response in IBD bears a number of similarities to that seen in rheumatoid arthritis, where TNF-α, IL-1 and IL-6 are prominent factors in synovial inflammation.[4] In both diseases, cytokine and acute phase protein levels are elevated, angiogenesis is up-regulated and endothelial dysfunction can be demonstrated [4, 5, 6] These similarities also exist with other diseases with an underlying inflammatory basis, such as psoriasis[7] and diabetic nephropathy.[8]

There is a well-described association between rheumatoid arthritis (RA) and increased cardiovascular mortality, mainly as a result of ischaemic heart disease (IHD).[9, 10] Atherosclerosis is increasingly recognized as a chronic inflammatory disorder, with evidence for endothelial dysfunction, and angiogenesis. More recently a causal relationship between IL-6R related pathways and coronary heart disease has been established.[11]

There are sufficient shared characteristics to the inflammatory responses seen in IBD and RA to hypothesise that the recognized association between RA and IHD might also exist for IBD and IHD. Previous cohort studies have examined the risk of IHD in patients with IBD and have variously found no effect,[12] or a slight increased risk [13]. A large Danish cohort study [13] found a high risk of IHD diagnosis in the first year after IBD diagnosis, followed by a small but statistically significant increased risk in subsequent years, a risk which decreased among users of 5-aminosalicylic acid (5-ASA) preparations. In this study we investigated both the strength and direction of association between IBD and IHD (including its sub-entities myocardial infarction (MI) and angina) in a large British cohort.

## Methods

### Study Population

The study population for this retrospective cohort study consisted of around 2.1 million male and female patients with an active registration at practices contributing to the General Practice Research Database (GPRD, now accessed via the Clinical Practice Research Datalink, http://www.cprd.com/home/) between 1987 and 2009. For each subject with IBD, a further 4 subjects were identified and matched for age and sex but without IBD. All cohort members were followed from the start date (i.e. the date their GPRD record began) until the earliest occurrence of one of the following endpoints: recorded code of IHD, death, date of exit from GPRD registered GP practice, or end of study period (31.12.2009). Patients with less than (1) 2 years up-to-standard enrolment with the GP or (2) 1 year of computerized prescription history or (3) no health contacts during their complete period of follow-up were excluded from the cohort. Patients whose date of death or date of transfer out of a GPRD-registered practice preceded date of IBD diagnosis were excluded from the cohort. Patients <15 years of age at entry were excluded.

### Disease definitions

We identified all male and female patients with a first diagnosis of IBD who met the following criteria: a first record of ulcerative colitis (UC), or Crohn’s Disease (CD), or inflammatory bowel disease (not otherwise specified) (IBD (nos)) recorded on the database since 1987; patients with ICD-10 Read Codes (K50-K51) records of both UC and IBD were coded as UC; CD plus IBD were coded as CD; individual records which included both UC and CD were included only in analyses of IBD (nos). Prevalent IBD cases on 01.01.1987 were excluded. The index date was defined as the date of first recording of IBD. Lifetime IBD severity was determined using an algorithm based on IBD drug-use (5-ASAs, corticosteroids, and immunosuppressants) and surgical intervention (colectomy/stomata) developed by team members with clinical and research expertise in IBD.

Age, sex and practice matched controls were allocated the same index date as their matched case. Because IHD has not been subjected to the same specific validation studies as IBD, our operational definition was developed using advice from clinicians with special interest in cardiovascular disease to include the following: Read Codes for IHD (to include myocarditis; myocardial infarction; acute coronary syndrome; angina; confirmation by exercise treadmill or referral to cardiologist; current prescriptions for glyceryl trinitrate; isosorbide mononitrate; isosorbide dinitrate; nicorandil; ivabradine) Coronary Heart Disease (CHD) was considered for the purpose of this study to be synonymous with IHD and Read Codes for coronary heart disease (including coronary artery disease) were included.

### Ethics Statement

Data was accessed within limits set out by the Medical Research Council licence agreement for academic access with Medical Research Ethics Committee ethical approval. The proposal was approved by the Independent Scientific Advisory Committee of the GPRD (protocol number 10_003).

### Analysis

Descriptive analysis of baseline characteristics and exploration of the direction of association between IBD and IHD was conducted using the whole cohort of patients aged over 15 years (COHORT A: n = 94,898). Subsequent analysis of the *a priori* hypothesis, that IHD followed IBD, was conducted on a subset which included cases of IHD following IBD and their associated matched controls (COHORT B: n = 77540).

The relative risks of ischaemic heart disease (IHD) and its sub-entities myocardial infarction (MI) and angina), as well as CHD were estimated as hazard ratios (HRs) for patients with IBD compared to non-IBD patients, using Cox proportional hazard models. Models were subsequently adjusted for age, gender, socio-economic status (using general practice postcode as a proxy), comorbidity and concomitant drug use, IBD disease severity, and smoking status. We carried out both forward and backward stepwise covariate selection within the cox proportional hazard model. All covariates were initially included with entry testing based on the significance of the score statistic, and removal testing based on the probability of a likelihood-ratio statistic based on conditional parameter estimates. Models were tested for interactions between fitted variables. Precision of estimates is reported using 95% confidence intervals. Data management was performed using Stata 10IC. Cox regression proportional hazard computations and Kaplan-Meier survival analysis were conducted using SPSS 21.

## Results

### Baseline cohort characteristics

**Fig 1:**
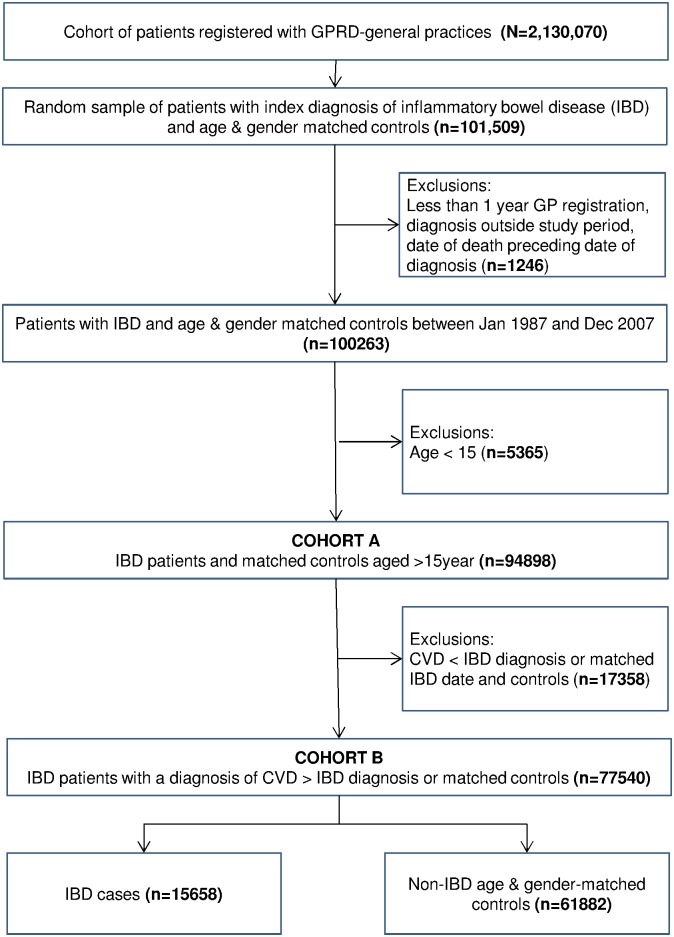
Cohort Flowchart. Selection of patients into two cohorts for analysis: COHORT A for descriptive analysis to examine the temporal relationship between IBD and IHD, and COHORT B for sub-group analysis of IHD cases which occurred following IBD diagnosis.

Data were examined from electronic primary care records from 221 general practices across the UK from patients with a first diagnosis of IBD recorded from 1st January 1987 to 31st December 2009 (Fig 1). Following exclusions, data from COHORT A (patients with IBD and matched controls aged over 15 years) was analysed to understand baseline characteristics and exploration of the direction of association between IBD and IHD (COHORT A: n = 94898, Tables 1 and 2). A subset of patients with IHD following IBD and their associated matched controls (COHORT B: n = 77540) was analysed to examine baseline characteristics and the strength of association between IBD and IHD (Tables 3–5). The mean follow up period was 6.8 years (SD 5.9). All-cause mortality during follow-up was 6353 (6.7%).

**Table 1:** COHORT A characteristics comparing non-IBD controls with IBD cases (≥15 years).

Characteristics	Non-IBD (n (%))	Crohn’s Disease (n (%))	Ulcerative Colitis(n (%))	IBD (NOS) (n (%))	P^6^
**Total number**	75735 (79.8%)	4620 (4.9%)	12397 (13.1%)	2146 (2.2%)	
**Male**	36028 (47.6%)	1991 (43.0%)	5883 (47.5%)	913 (42.5%)	<0.001
**Age**					
** 15-34y**	21759 (28.7%)	1816 (39.3%)	2960 (23.9%)	699 (32.6%)	
** 35-49y**	19365 (25.6%)	1115 (24.1%)	3182 (25.7%)	591 (27.5%)	
** 50-64y**	17089 (22.6%)	886 (19.2%)	3036 (24.5%)	421 (19.6%)	
** 65+y**	17522 (23.1%)	803 (17.4%)	3219 (26.0%)	435 (20.3%)	
***Mean age at entry (SD)***	48.8 (SD 18.9)	44.2 (SD 18.9)	50.9 (SD 18.5)	46.9 (SD 19.0)	<0.001
**IBD severity** ^1^					
** Mild**	-	2325 (50.3%)	8141 (65.7%)	1424 (66.4%)	
** Moderate**	-	1572 (34.0%)	3220 (26.0%)	594 (27.7%)	
** Severe**	-	723 (15.7%)	1036 (8.4%)	128 (6.0%)	
**Smoker** ^2^					
** Never**	27635 (54.4%)	540 (60.1%)	1429 (68.1%)	232 (66.7%)	
** Ever**	23120 (45.6%)	358 (39.9%)	669 (31.9%)	116 (33.3%)	<0.001
**BMI** ^3^					
** <10**	4 (0.0%)	1 (0.0%)	1 (0.0%)	0 (0.0%)	
** 11–15**	197 (0.3%)	29 (0.8%)	37 (0.4%)	6 (0.3%)	
** 16–18.49**	1415 (2.3%)	195 (5.2%)	259 (2.6%)	51 (2.9%)	
** 18.5–24.9**	25499 (40.8%)	1769 (47.0%)	4220 (41.8%)	769 (43.2%)	
** 25–30**	22523 (36.0%)	1130 (30.0%)	3557 (35.3%)	622 (34.9%)	
** >30**	12878 (20.6%)	645 (17.1%)	2014 (20.0%)	333 (18.7%)	
***Mean (SD)***	26.5 (SD 5.4)	25.4 (SD 5.5)	26.3(SD 5.3)	26.2 (SD 5.68)	0.198
**Practice IMD** ^4^					
** 0**	4758 (20.6%)	271 (14.2%)	811 (16.1%)	108 (13.7%)	<0.001
** 1**	4797 (20.7%)	391 (20.5%)	1010 (20.1%)	160 (20.3%)	<0.001
** 2**	3451 (14.9%)	374 (19.6%)	967 (19.2%)	138 (17.5%)	<0.001
** 3**	6320 (27.3%)	443 (23.2%)	1193 (23.7%)	205 (26.1%)	<0.001
** 4**	3836 (16.6%)	431 (22.6%)	1050 (20.9%)	176 (22.4%)	<0.001
**Comorbidities**					
** Rheumatoid Arthritis**	1180 (1.6%)	101 (2.2%)	321 (2.6%)	43 (2.0%)	<0.001
** Diabetes type 2**	4869 (6.4%)	214 (4.6%)	849 (6.9%)	108 (5.0%)	0.108
** Diabetes type 1**	599 (0.8%)	42 (0.9%)	153 (1.2%)	22 (1.0%)	<0.001
** Chronic kidney disease**	3567 (4.7%)	156 (3.4%)	590 (4.8%)	91 (4.2%)	0.046
**Contraceptive use**	7954 (10.5%)	1004 (21.7%)	1757 (14.2%)	459 (21.4%)	<0.001
**IBD drug use** ^5^					
** 5-Aminosalicylic acid**	445 (0.6%)	3264 (70.7%)	9085 (73.3%)	1380 (64.3%)	<0.001
** Oral Corticosteroids**	10178 (13.4%)	3140 (68.0%)	8046 (64.9%)	1243 (57.9%)	<0.001
** Azathioprine/methotrexate**	650 (0.9%)	1456 (31.5%)	1983 (16.0%)	326 (15.2%)	<0.001
** Tumour necrosis factor α antagonists**	58 (0.8%)	14 (0.3%)	111 (0.9%)	16 (0.8%)	<0.001
**Charlson Score** ^5^					
** 0**	43422 (70.2%)	2458 (62.6%)	5930 (59.7%)	1116 (62.1%)	
** 1**	10430 (16.9%)	908 (23.1%)	2204 (22.2%)	414 (23.0%)	
** 2**	4190 (6.8%)	277 (7.1%)	877 (8.8%)	132 (7.3%)	
** 3**	2109 (3.4%)	146 (3.7%)	468 (4.7%)	70 (3.9%)	
** 4**	834 (1.3%)	65 (1.7%)	210 (2.1%)	30 (1.7%)	
** 5**	438 (0.7%)	23 (0.6%)	89 (0.9%)	9 (0.5%)	
** 6 or more**	459 (0.7%)	51 (1.3%)	154 (1.6%)	27 (1.5%)	<0.001
**Major abdominal surgery** ^6^	0 (0.0%)	635 (16.2%)	712 (7.2%)	102 (5.7%)	<0.001
** Stoma**	0 (0.0%)	222 (5.7%)	460 (4.6%)	51 (2.8%)	<0.001

^1^Estimated using IBD-drug use and surgical interventions during follow-up as proxies (see text for further detail)

^2^ 43% of patients had missing data

^**3**^ 17.4% of patients had missing data

^4^Index of Multiple Deprivation (IMD) based on practice post-code. Quintile 0 is the least deprived, quintile 4 is the most deprived. 67.5% of patients had missing data.

^5^Ever in patient record

^6^ Non-IBD compared to IBD, comparison of binary variables by adjusted χ2 test; continuous variables by Student’s t-test; multiple category variables by χ2 test adjusted for trend.

**Table 2:** Mean age at diagnosis of ischaemic heart disease comparing patients with and without IBD (COHORT A)*.

	Mean age at diagnosis of heart disease (SD, 95% CI^1^, p)			
**All heart disease** ^2^				
CD cases	65.2	13.4	-3.8–1.6	0.435
CD controls	66.3	3.5	1-3.8–1.7	
UC	65.8	11.7	-2.6–-0.2	**0.021**
UC controls	67.4	12.6	-2.8–0.3	
**Angina**				
CD cases	62.0	11.3	-7.8–0.0	**0.052**
CD controls	65.9	12.6	-7.5–-0.2	
UC	64.5	11.0	-4.4–-0.7	**0.008**
UC controls	67.0	12.3	-4.4–-0.8	
**Coronary Heart Disease**				
CD cases	65.7	12.7	-4.7–2.7	0.589
CD controls	66.7	12.5	-4.8–2.8	
UC	65.7	11.9	-3.9–-0.4	**0.015**
UC controls	67.9	11.7	-3.9–-0.4	
**Ischaemic Heart Disease**				
CD cases	68.0	13.6	-2.9–4.8	0.619
CD controls	67.0	12.9	-3.0–5.0	
UC	67.3	11.2	-2.4–1.1	0.460
UC controls	68.0	12.5	-2.3–1.0	
**MI**				
CD cases	67.5	13.4	-4.8–5.6	0.877
CD controls	67.1	14.3	-4.7–5.5	
UC	66.3	12.1	-4.2–0.9	0.193
UC controls	67.9	13.3	-4.2–0.7	

^1^ 95% Confidence Interval of the Difference between patients with and without IBD using Student’s T-Test

^2^incorporates IHD, CHD, angina and MI

*Analysis excludes cases of IHD diagnosed prior to IBD diagnosis.

**Table 3:** COHORT B characteristics comparing non-IBD controls with IBD cases*.

Characteristics	Non-IBD (n(%))	Crohn’s Disease (n(%))	Ulcerative Colitis (n(%))	IBD (NOS) (n(%))	P^7^
**Total number**	**61882 (79.8%)**	**3928 (5.1%)**	**9932 (12.8%)**	**1798 (2.3%)**	
**Male**	29895 (48.3%)	1686 (42.9%)	4667 (47.0%)	758 (42.2%)	<0.001
**Age**					
** 15-34y**	21700 (35.1%)	1815 (46.2%)	2948 (29.7%)	697 (38.8%)	
** 35-49y**	18868 (30.4%)	1087 (46.2%)	3102 (29.7%)	574 (31.9%)	
** 50-64y**	13192 (21.3%)	667 (17.0%)	2354 (23.7%)	341 (19.0%)	
** 65+y**	8122 (13.1%)	359 (9.1%)	1528 (15.4%)	186 (10.3%)	
***Mean age at entry (SD)***	*46*.*6 (SD 20*.*6)*	*39*.*9 (SD 16*.*6)*	*46*.*2 (SD 16*.*8)*	*42*.*3 (SD 16*.*6)*	*0*.*495*
**IBD severity** ^1^					
** Mild**	-	1952 (49.7%)	6547 (65.9%)	1192 (66.3%)	
** Moderate**	-	1338 (34.1%)	2532 (25.5%)	502 (27.9%)	
** Severe**	-	638 (16.2%)	853 (8.6%)	104 (5.8%)	
**Smoker** ^2^					
** Never**	27635 (54.4%)	540 (60.1%)	1429 (68.1%)	232 (66.7%)	
** Ever**	23120 (45.6%)	358 (39.9%)	669 (31.9%)	116 (33.3%)	<0.001
**BMI** ^**3**^					
** <10**	4 (0.0%)	0 (0.0%)	0 (0.0%)	0 (0.0%)	
** 11–15**	143 (0.3%)	27 (0.8%)	23 (0.3%)	3 (0.2%)	
** 16–18.49**	1151 (2.2%)	180 (5.4%)	198 (2.4%)	44 (2.9%)	
** 18.5–24.9**	21922 (41.3%)	1579 (47.7%)	3611 (43.0%)	668 (43.3%)	
** 25–30**	18909 (35.6%)	963 (29.1%)	2901 (34.5%)	538 (34.9%)	
** >30**	10948 (20.6%)	558 (16.9%)	1671 (19.9%)	288 (18.7%)	
***Mean (SD)***	*26*.*5 (SD 5*.*4)*	*25*.*3 (SD 5*.*4)*	*26*.*3 (SD 5*.*3)*	*26*.*2 (SD 5*.*4)*	<0.001
**Practice IMD** ^4^					
**0**	3743 (21.0%)	242 (14.9%)	650 (16.3%)	86 (13.4%)	<0.001
**1**	3722 (20.8%)	335 (20.6%)	792 (19.9%)	125 (19.4%)	<0.001
**2**	2574 (14.4%)	310 (19.0%)	774 (19.4%)	112 (17.4%)	<0.001
**3**	4827 (27.0%)	384 (23.6%)	948 (23.8%)	164 (25.5%)	<0.001
**4**	2996 (16.8%)	358 (22.0%)	821 (20.6%)	157 (24.4%)	<0.001
**Comorbidities**					
** Rheumatoid Arthritis**	826 (1.3%)	75 (1.9%)	234 (2.4%)	29 (1.6%)	<0.001
** Diabetes type 2**	3040 (4.9%)	136 (3.5%)	532 (5.4%)	70 (3.9%)	0.309
** Diabetes type 1**	421 (0.7%)	21 (0.5%)	95 (1.0%)	16 (0.9%)	0.032
** Chronic kidney disease**	2022 (3.3%)	103 (2.6%)	365 (3.7%)	55 (3.1%)	0.653
** Contraceptive use**	7894 (12.8%)	999 (25.4%)	1730 (17.4%)	456 (25.4%)	<0.001
**IBD drug use**					
** 5-Aminosalicylic acid**	327 (0.5%)	2811 (71.6%)	7352 (74.0%)	1160 (64.5%)	<0.001
** Oral Corticosteroids**	7455 (12.0%)	2665 (67.8%)	6415 (64.6%)	1038 (57.7%)	<0.001
** Azathioprine/methotrexate**	474 (0.8%)	1320 (33.6%)	1709 (17.2%)	301 (16.7%)	<0.001
** Tumour necrosis factor α antagonists**	43 (0.1%)	12 (0.3%)	98 (1.0%)	12 (0.7%)	<0.001
**Charlson Score** ^5^					
** 0**	43422 (70.2%)	2458 (62.6%)	5930 (59.7%)	1116 (62.1%)	
** 1**	10430 (16.9%)	908 (23.1%)	2204 (22.2%)	414 (23.0%)	
** 2**	4190 (6.8%)	277 (7.1%)	877 (8.8%)	132 (7.3%)	
** 3**	2109 (3.4%)	146 (3.7%)	468 (4.7%)	70 (3.9%)	
** 4**	834 (1.3%)	65 (1.7%)	210 (2.1%)	30 (1.7%)	
** 5**	438 (0.7%)	23 (0.6%)	89 (0.9%)	9 (0.5%)	
** 6 or more**	459 (0.7%)	51 (1.3%)	154 (1.6%)	27 (1.5%)	<0.001
**Major abdominal surgery** ^6^	0 (0.0%)	635 (16.2%)	712 (7.2%)	102 (5.7%)	<0.001
** Stoma**	0 (0.0%)	222 (5.7%)	460 (4.6%)	51 (2.8%)	<0.001

^1^Estimated using IBD-drug use as proxies (see text for further detail)

^2^30.2% of patients had missing data during follow-up (index date to IHD date or end of follow-up).

^3^14.2% of patients had missing data during follow-up (BMI record nearest to IHD date (+-12 months)).

^4^Index of Multiple Deprivation (IMD) based on practice post-code. Quintile 0 is the least deprived, quintile 4 most deprived.

^5^Charlson index [17] is a validated index of comorbidity taking into account both the number and severity of comorbidities. 0 is no comorbidities. 6 or more is a very high score indicating a high number of cormorbidities and/or severe comorbidities. For example AIDS and metastatic tumour each score 6.

^6^Colectomy during follow-up (index date to IHD date or end of follow-up).

^7^Non-IBD compared to IBD, comparison of binary variables by adjusted χ2 test; continuous variables by Student’s t-test; multiple category variables by χ2 test adjusted for trend

*Analysis excludes cases of IHD diagnosed prior to IBD diagnosis.

**Table 4:** Incidence of IHD among cases and controls (COHORT B)*.

	Non-IBD	Crohn’s Disease	Ulcerative Colitis	IBD (NOS)
**All heart disease** ^1^	2343 (3.8%)	117 (3.0%)	434 (4.4%)	56 (3.1%)
Angina	1172 (1.9%)	48 (1.2%)	196 (2.0%)	33 (1.8%)
CHD	1251 (2.0%)	55 (1.4%)	215 (2.2%)	26 (1.4%)
IHD	1162 (1.9%)	57 (1.5%)	238 (2.4%)	32 (1.8%)
MI	673 (1.1%)	36 (0.9%)	131 (1.3%)	20 (1.1%)

^1^incorporates IHD, CHD, angina and MI

*Analysis excludes cases of IHD diagnosed prior to IBD diagnosis.

**Table 5:** Unadjusted hazard ratios for cardiovascular disease comparing IBD drug users* and non-users (COHORT B).

	HR (95%CI), p unadjusted comparing IBD and non-IBD patients	HR (95%CI), p unadjusted comparing users of Azathioprine/methotrexate^1^ and non-users	HR (95%CI), p unadjusted comparing users of Tumour necrosis factor α antagonists^1^ and non-users	HR (95%CI), p unadjusted comparing users of 5ASAs^1^ and non-users	HR (95%CI), p unadjusted comparing users of Corticosteroids^1^ and non-users
**Cardiovascular disease**					
IBD	1.2 (1.1–1.3), p<0.001	1.0 (0.8–1.1) p = 0.614	1.5 (0.8–2.8) p = 0.256	1.2 (1.1–1.3) p = <0.001	1.8 (1.6–1.9) p<0.001
CD	1.2 (1.0–1.5), p = 0.059	0.8 (0.6–1.2) p = 0.327	1.4 (0.2–9.8) p = 746	1.1 (0.9–1.4) p = 0.322	1.7 (1.4–2.0) p<0.001
UC	1.2 (1.1–1.4) p<0.001	1.1 (0.9–1.4) p = 0.324	1.0 (0.4–2.4) p = 0.997	1.2 (1.1–1.4) p = 0.001	1.8 (1.6–1.9) p<0.001
**Angina**					
IBD	1.1 (1.0–1.2) p = 0.235	1.0 (0.7–1.2) p = 0.653	2.0 (0.9–4.4) p = 0.095	1.1 (0.9–1.2) p = 0.449	1.8 (1.6–2.0) p<0.001
CD	1.0 (0.8–1.4) p = 0.836	0.7 (0.4–1.2) p = 0.141	0.1 (0.0–241) p = 0.702	1.1 (0.8–1.5) p = 0.654	2.0 (1.6–2.6) p<0.001
UC	1.1 (0.9–1.2) p = 0.428	1.2 (0.9–1.6) p = 0.287	1.2 (0.4–3.7) p = 0.753	1.0 (0.9–1.2) p = 0.703	1.7 (1.5–2.0) p<0.001
**Coronary Heart Disease**					
IBD	1.1 (1.0–1.3) p = 0.081	1.2 (0.9–1.4) p = 0.210	1.9 (0.8–4.2) p = 0.128	1.2 (1.0–1.4) p = 0.022	1.7 (1.6–1.9) p<0.001
CD	1.1 (0.8–1.5) p = 0.459	1.0 (0.7–1.6) p = 0.929	2.7 (0.4–19.1) p = 0.326	1.2 (0.9–1.7) p = 0.250	1.9 (1.5–2.5) p<0.001
UC	1.1 (1.0–1.3) p = 0.108	1.3 (1.0–1.7) p = 0.038	1.5 (0.6–4.1) p = 0.404	1.2 (1.0–1.4) p = 0.093	1.7 (1.5–1.9) p<0.001
**Ischaemic Heart Disease**					
IBD	1.3 (1.2–1.5) p<0.001	1.0 (0.7–1.2) p = 0.688	1.9 (0.9–4.3) p = 0.107	1.3 (1.2–1.5) p<0.001	1.8 (1.6–2.0) p<0.001
CD	1.2 (1.0–1.6) p = 0.180	0.8 (0.5–1.3) p = 0.375	3.0 (0.4–21.6) p = 0.268	1.3 (0.9–1.8) p = 0.145	1.9 (1.5–2.4) p<0.001
UC	1.3 (1.1–1.5) p<0.001	1.1 (0.9–1.5) p = 0.388	1.2 (0.4–3.6) p = 0.789	1.3 (1.1–1.5) p = 0.001	1.8 (1.6–2.0) p<0.001
**MI**					
IBD	1.3 (1.1–1.5) p = 0.002	1.1 (0.8–1.5) p = 0.464	1.1 (0.3–4.5) p = 0.878	1.4 (1.2–1.7) p<0.001	1.8 (1.5–2.0) p<0.001
CD	1.2 (0.8–1.7) p = 0.369	0.6 (0.3–1.3) p = 0.215	0.05 (0.0–485) p = 0.749	1.1 (0.7–1.7) p = 0.652	1.5 (1.1–2.0) p = 0.021
UC	1.4 (1.1–1.6) p = 0.004	1.5 (1.0–2.1) p = 0.036	0.7 (0.1–5.1) p = 0.737	1.5 1.2–1.8) p<0.001	1.9 (1.6–2.3) p<0.001

^1^Drug users with and without IBD were included in this analysis

*Analysis excludes cases of IHD diagnosed prior to IBD diagnosis.

### Temporal relationship between IBD and IHD (COHORT A)

**Fig 2:**
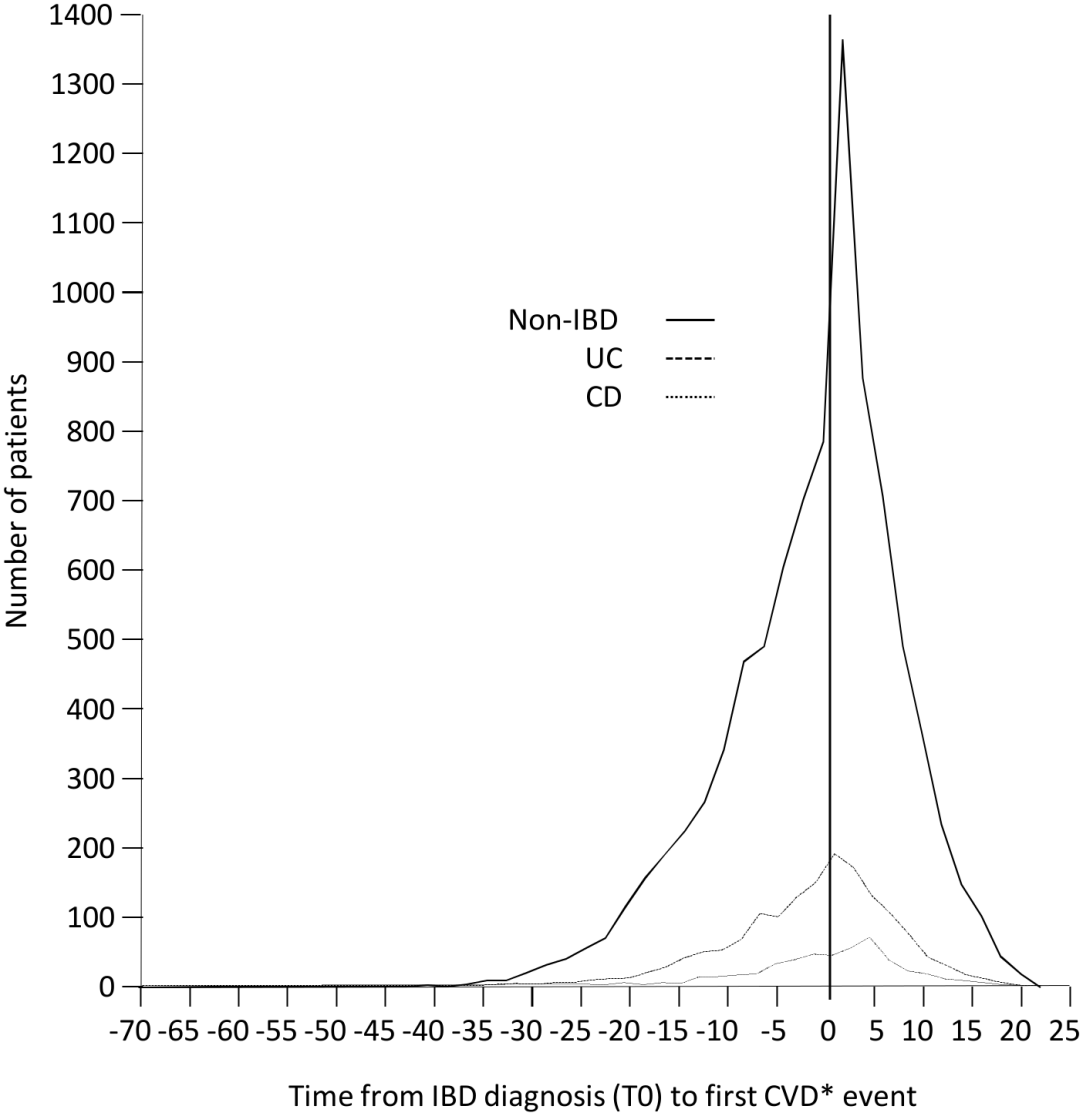
Time from IBD diagnosis (T0) to first IHD event according to IBD type. Total number of patients showing time from IBD diagnosis (T0) to the first cardiovascular event (incorporating IHD, CHD, angina and MI).

Of 19163 IBD cases, a total of 2220 (11.6%) had an IHD diagnosis ever, compared with 6504 (8.6%) of those without IBD. Of these IHD diagnoses, 4494 (51.5%) occurred prior to IBD diagnosis (1404 (63.2%) of IBD cases and 3090 (47.5%) of controls) (Fig 2). According to IBD type, of 12,397 cases of Ulcerative Colitis, 1557 (12.6%) had an IHD diagnosis ever, and of these, 980 (62.9%) occurred prior to IBD diagnosis; while of 4620 cases of Crohn’s Disease, 445 (9.6%) had an IHD diagnosis ever, of which 284 (63.8%) occurred prior to IBD diagnosis (S1–S7 Figs). Similar temporal trends were seen in angina, MI and CHD. Sub-group analysis of IHD cases diagnosed during or after the year 2004 also showed similar temporal trends. An increased incidence of IHD, angina, MI, and CHD diagnoses was seen in the first year after IBD diagnosis (S1–S6 Figs) but the mean age of IHD diagnosis comparing IBD cases with non IBD cases was not statistically different apart from those with Ulcerative Colitis (UC) who were slightly younger at diagnosis of angina and coronary heart disease (Table 2; S7 Fig). Of those developing IHD following an IBD diagnosis, patients with UC were at higher risk of a diagnosis of IHD (unadjusted HR 1.3, 95% CI 1.1–1.5, p<0.001) or MI (unadjusted HR 1.4, 95% CI 1.1–1.6, p = 0.004). Adjustment for a range of factors including age and gender showed interaction between variables; models did not reach statistical significance and thus are not reported here.

### Subset characteristics (COHORT B: IBD preceding IHD)

A total of 17358 patients were excluded from subgroup analysis where IHD preceded IBD or, for controls, the matched IBD date (associated cases/controls were also excluded in order to protect matching of the resulting subset) (Fig 1). Subset characteristics (n = 77540) were comparable to those of the whole cohort (Table 3). Overall, the incidence of IHD following IBD diagnosis (or matched control date) was similar to those without IBD (3.8% vs 3.9% p = 0.001). The incidence of IHD following IBD diagnosis (or matched control) was slightly higher in those with Ulcerative Colitis and lower in those with Crohn’s disease compared with patients without IBD (4.4% vs 3.0% vs 3.8%, p = 0.042). This trend was also seen in angina, MI and CHD (Table 4). In unadjusted analysis, Ulcerative Colitis was associated with a small but statistically significant increased risk of IHD, and MI (HR 1.3, 95% CI 1.1–1.5, p<0.001; HR 1.4, 95% CI 1.1–1.6, p = 0.004 respectively) (Table 5). Use of 5ASAs did not alter this risk but users of corticosteroids were at significantly greater risk of IHD and its subtypes.

In order to examine the subset in a way that was consistent with previous research,[13] the incidence rate ratios (IRR) for IHD were calculated (Table 6), comparing COHORT B, the partitioned subset, (i.e. IHD>IBD) with COHORT A (i.e. IHD event ever). The IRR for COHORT A was 2.1 in the first year of IBD diagnosis, with an overall IRR of 1.8. In the partitioned subset (COHORT B), the IRR in the first year of IBD diagnosis was lower (1.26) and similarly low overall IRR (1.22).

**Table 6:** Incidence Rate Ratios (IRR) comparing whole group analysis with sub-set*.

	Whole dataset (COHORT A)		Sub-set (COHORT B)	
	Overall IRR (95% CI)	IRR in 1^st^ year > IBD diagnosis(95% CI)	Overall IRR (95% CI)	IRR in 1^st^ year > IBD diagnosis (95% CI)
**All IHD**	1.8 (1.72–1.89)	2.1 (1.98–2.22)	1.2 (1.12–1.33)	1.3 (1.07–1.48)

*Analysis excludes cases of IHD diagnosed prior to IBD diagnosis.

## Discussion

This is the first study to fully examine both the strength and direction of association between development of ischaemic heart disease (IHD) and IBD. An increased incidence of IHD, angina, MI, and CHD diagnoses was seen in the first year after IBD diagnosis but the mean age of diagnosis comparing IBD cases with non IBD cases was not statistically different, apart from those with Ulcerative Colitis (UC) who were slightly younger at diagnosis of angina and coronary heart disease. Of those developing IHD following an IBD diagnosis, patients with UC were at slightly higher risk of a diagnosis of IHD or MI although results were not significant after adjustment for the role of covariates. Most notably, we found that although overall prevalence of IHD was higher in those with IBD, in the majority of cases the diagnosis of IHD actually predated that of IBD.

This is the largest British cohort study to examine this issue. It used the General Practice Research Database which is an appropriate and reliable resource for IBD research, having been used for at least 19 studies published in peer-reviewed journals since 2000. The diagnostic reliability of IBD diagnosis and the year of IBD diagnosis in the GPRD has been confirmed in a recent validation study.[14] Other GPRD validation studies have confirmed reliable recording of cardiovascular disease and other disease groups.[15, 16] The risk of selection bias in this study was low given that GPRD data is collected from a wide cross-section of general practices from across the UK. The prevalence of CD is slightly lower compared to UC than would be expected in a UK population [17]. However, the cohort was a random selection taken from 2 million records and the difference is unlikely to reflect systematic sampling bias. We were careful to exclude from our analysis patients with chronic kidney disease and diabetes mellitus, since both are associated with raised inflammatory markers and could have confounded our results.

As others have highlighted [13], there is a risk of ascertainment bias because IBD patients may have more frequent contact with health care professionals and are therefore monitored more closely, potentially raising the chance of earlier detection of IHD in this group. The fact that patients with UC were at slightly higher risk of IHD and MI could potentially be a selection effect on a conditioned group or simply an artefact of multiple hypotheses in this hypothesis generating study. We sought to overcome this by designing the study to examine the direction of association and clearly delineating prevalent and incident IHD cases. In addition, we allowed adjustment for the effect of comorbidities in both cases and controls by using the Charlson index [18] as a composite measure of comorbidity within multiple analyses, all of which demonstrated no difference between groups.

The temporal relationship between IBD and IHD may be weakened by differences with which the diagnosis of each is recorded in the GPRD. IHD is a diagnosis made in the majority of cases fairly soon after the onset of symptoms, because of an event requiring admission to hospital (MI, acute coronary syndrome). Nevertheless, among those with stable angina, nearly half have symptoms for 6 months or more before diagnosis.[19] For IBD the time to reach a diagnosis may be longer. A Swiss cohort study of 1591 patients with IBD estimated the median interval from first presentation to a GP to diagnosis as 4 months (IQR 0–18) for CD and 1 month (0–5) for UC.[20] Independent risk factors for delayed diagnosis included younger age (<40) in Crohn’s disease and male sex in ulcerative colitis.[20]

It is possible that IHD cases may have been missed or incorrectly coded within GPRD. Unrecorded diagnoses are increasingly unlikely within UK general practice due to policy and fiscal incentives to assess, monitor and treat cardiovascular disease,[21] although historical records pre-dating these incentives are more likely to have unrecorded IHD diagnoses. Sub-group analysis comparing diagnoses of IHD and its subtypes before and after these incentives (around the year 2004) demonstrate no differences either in temporal trends or in overall risk profiles, thus decreasing the likelihood of reporting bias. Incorrect sub-group coding of types of IHD is another possibility, partly as a result of lack of clinical consensus about definitional boundaries. For this reason, we conducted sub-group analysis on MI, angina and additional group analysis of cases coded as CHD and the fact that trends were entirely consistent with results for IHD goes some way to refuting the potential for this type of recording bias. Although GPRD prescribing and clinical data are robust and reliable, BMI and smoking data were incomplete due to the fact that, in the UK, many general practices do not routinely or systematically collect this data. Data on cholesterol levels were not requested. Thus the effect of these covariates on the association between IHD and IBD remains unknown. We were unable to reliably differentiate between remission and relapse phases, or to capture concomitant extraintestinal manifestations. These data would be recorded in secondary care but not routinely communicated to primary care in a way that would result in the event being routinely coded in the primary care record, and hence the GPRD dataset. The potential association between relapses and incidence of heart disease warrants further investigation.

This is the first known study to examine the temporal relationship between IHD and IBD and we found that the majority of IHD cases predated IBD diagnosis. Previous research has predominantly focused attention on incident cases of IHD occurring at or after diagnosis of IBD. It is notable that the overall risk of prevalent (i.e. whole cohort) IHD was almost identical to a recent Danish study [13] at 2.1 IRR, but that when this was limited to truly incident cases post IBD, the risk reduced to 1.2 IRR. IBD is triggered by a complex interaction between environmental factors, genetic predisposition, and an exaggerated immune response. Our findings suggest that the temporal relationship between IBD and IHD is against the concept of the latter being a long term consequence of IBD-related inflammation, as previously proposed [13]. Instead, the association with IHD is more plausibly explained by common mechanisms in the inflammatory pathway, or even a reverse causality. Our findings suggest that the assessment of a patient’s future risk of IHD should not be modified by the presence of IBD. Among patients who develop IHD, those who have raised cytokine levels, particularly of IL6 and TNF Alpha, may be at increased risk of subsequently developing IBD.

### Conclusions

By identifying and quantifying both the strength and direction of association between IBD and IHD, this study provides important insights into the burden of IHD in the IBD population. This is relevant for basic scientists exploring the temporal causal pathway, and clinicians who should be aware of the risks of IHD in patients with IBD and vice versa.

## Supporting Information

S1 Fig Time from IBD diagnosis (T0) to first CVD event by age. Total number of patients showing time from IBD diagnosis (T0) to the first cardiovascular event (incorporating IHD, CHD, angina and MI) according to IBD type and age categories.
(TIF)

S2 Fig Time from IBD diagnosis (T0) to first CVD event by IBD type and gender. Total number of patients showing time from IBD diagnosis (T0) to the first cardiovascular event (incorporating IHD, CHD, angina and MI) according to IBD type and gender categories.
(TIF)

S3 Fig Time from IBD diagnosis (T0) to first coronary heart disease event by IBD type. Total number of patients showing time from IBD diagnosis (T0) to the first Coronary Heart Disease (sub-group of CVD) event according to IBD type.
(TIF)

S4 Fig Time from IBD diagnosis (T0) to first Ischaemic Heart Disease event by IBD type. Total number of patients showing time from IBD diagnosis (T0) to the first Ischaemic Heart Disease (sub-group of CVD) event according to IBD type.
(TIF)

S5 Fig Time from IBD diagnosis (T0) to first angina event according to IBD type. Total number of patients showing time from IBD diagnosis (T0) to the first Angina (sub-group of CVD) event according to IBD type.
(TIF)

S6 Fig Time from IBD diagnosis (T0) to first Myocardial Infarction event according to IBD type Total number of patients showing time from IBD diagnosis (T0) to the first Myocardial Infarction (sub-group of CVD) event according to IBD type.
(TIF)

S7 Fig Age at CVD onset according to IBD type Total number of patients showing age at onset of Cardiovascular Disease (incorporating IHD, CHD, angina and MI) according to IBD type.
(TIF)
