# Supplementary figures and images for: Risk of Ischaemic Heart Disease in Patients with Inflammatory Bowel Disease: Cohort Study Using the General Practice Research Database
Source: PLoS One. 2015 Oct 13;10(10):e0139745. doi: 10.1371/journal.pone.0139745 (PMC4604089; doi:10.1371/journal.pone.0139745)

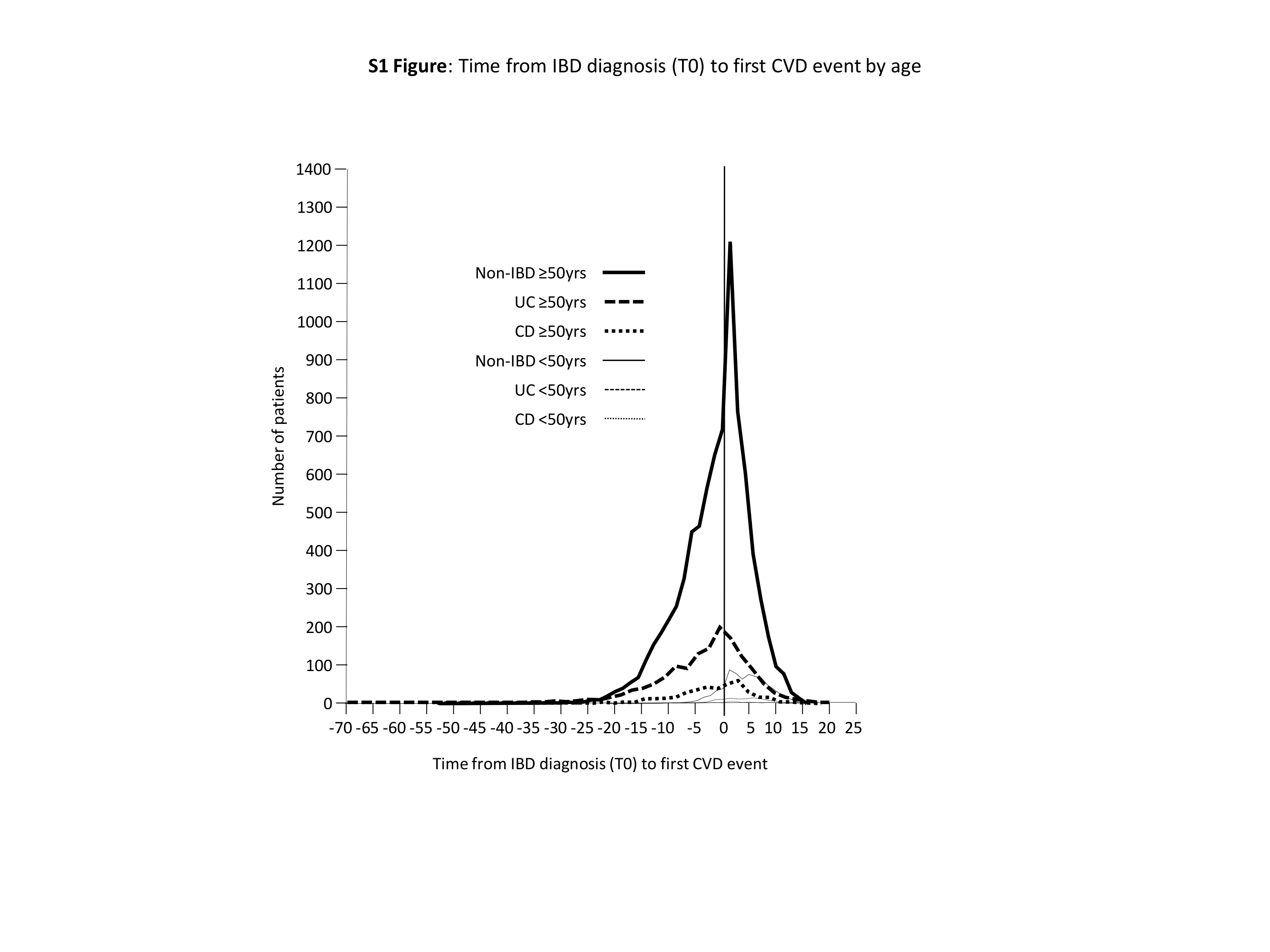

Supplement: S1 Fig — Total number of patients showing time from IBD diagnosis (T0) to the first cardiovascular event (incorporating IHD, CHD, angina and MI) according to IBD type and age categories. (TIF) [file pone.0139745.s001.tif]

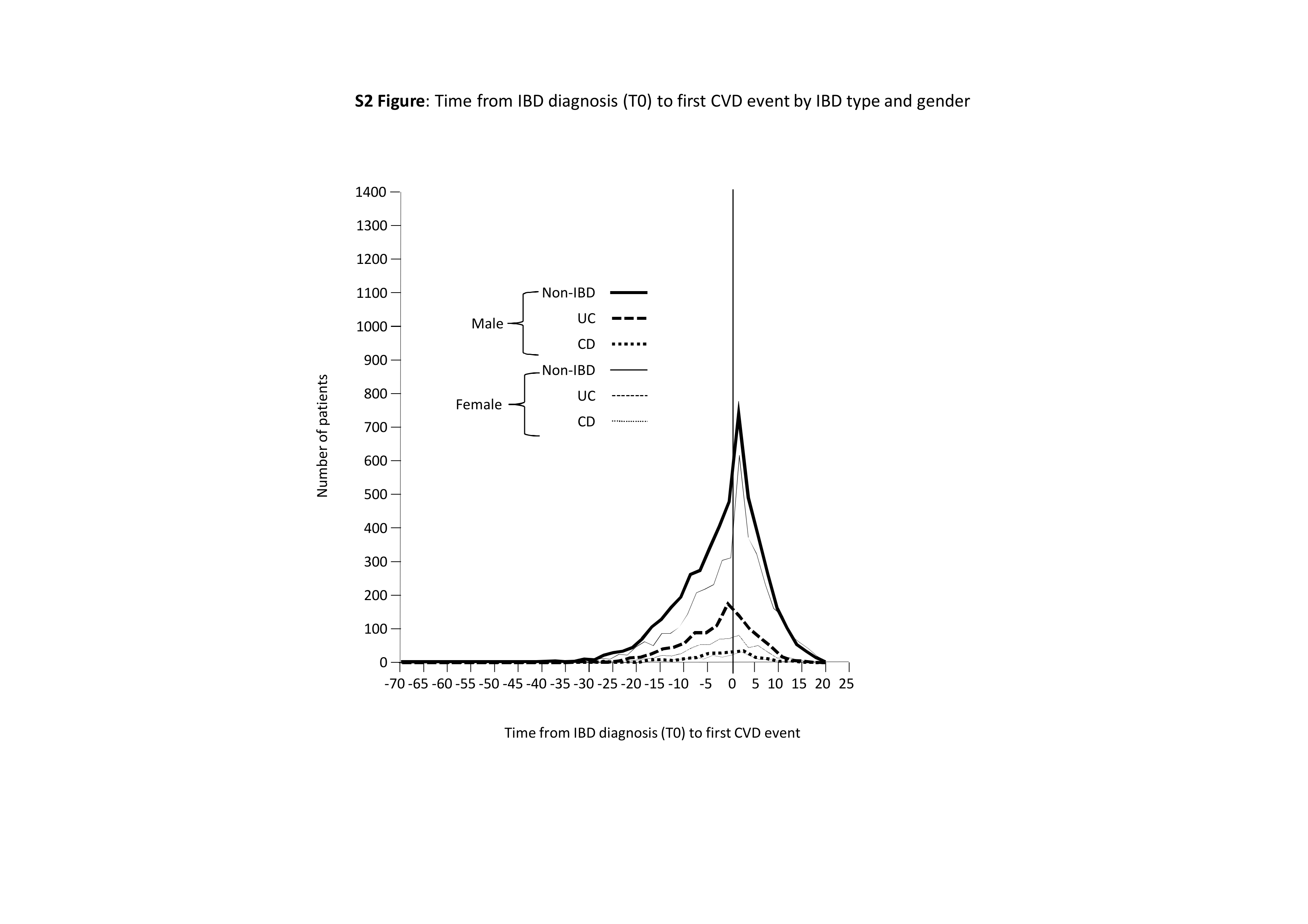

Supplement: S2 Fig — Total number of patients showing time from IBD diagnosis (T0) to the first cardiovascular event (incorporating IHD, CHD, angina and MI) according to IBD type and gender categories. (TIF) [file pone.0139745.s002.tif]

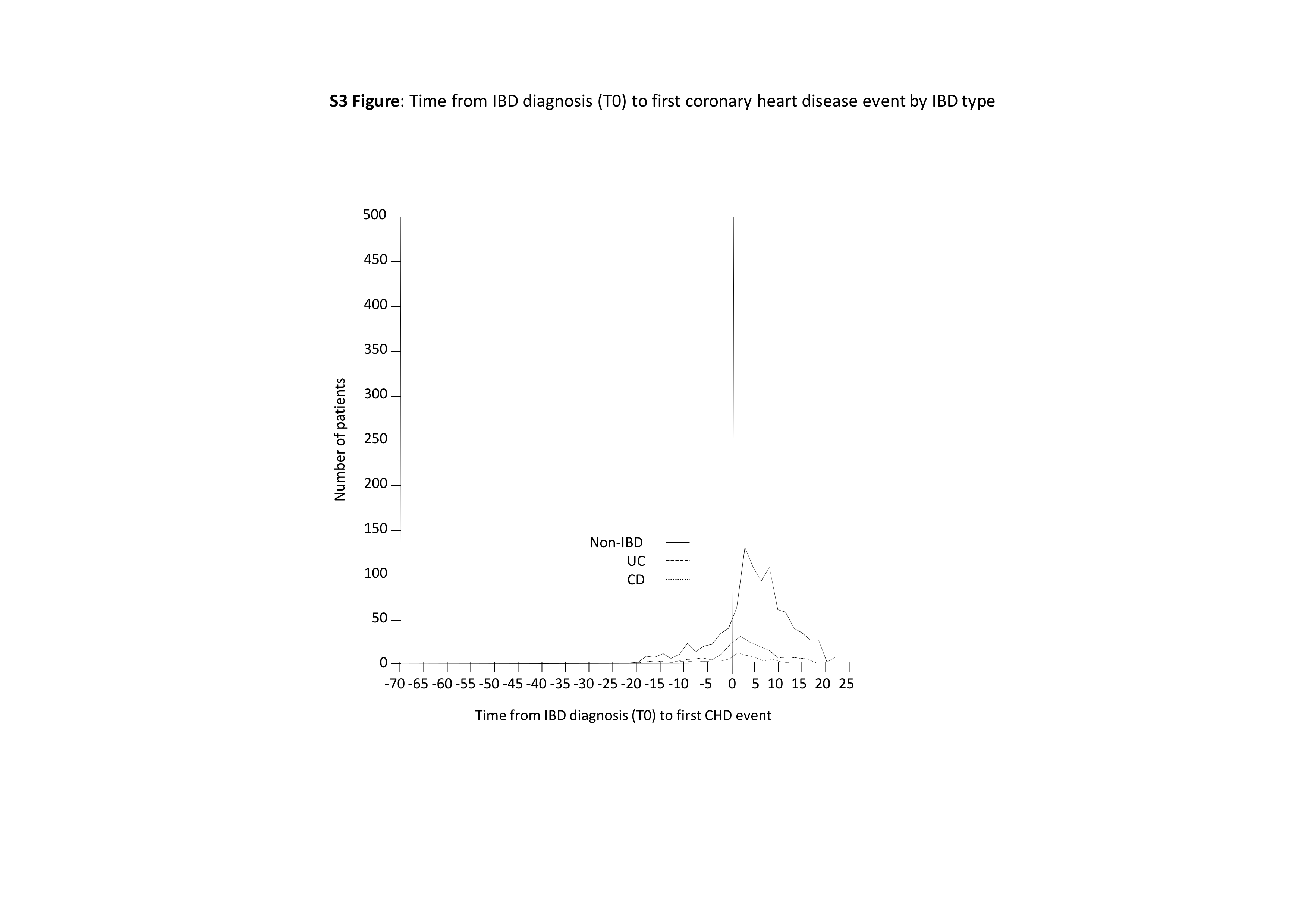

Supplement: S3 Fig — Total number of patients showing time from IBD diagnosis (T0) to the first Coronary Heart Disease (sub-group of CVD) event according to IBD type. (TIF) [file pone.0139745.s003.tif]

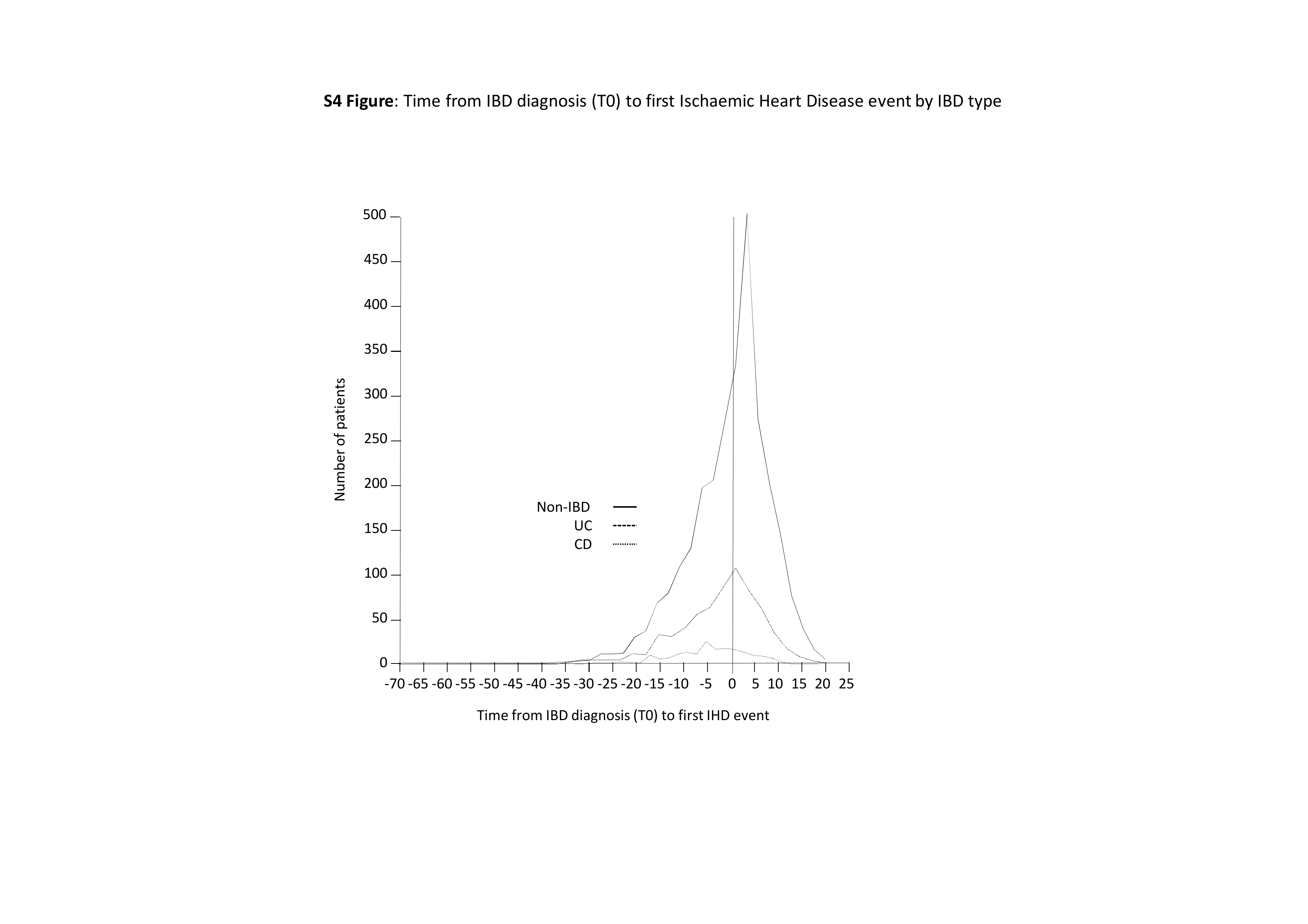

Supplement: S4 Fig — Total number of patients showing time from IBD diagnosis (T0) to the first Ischaemic Heart Disease (sub-group of CVD) event according to IBD type. (TIF) [file pone.0139745.s004.tif]

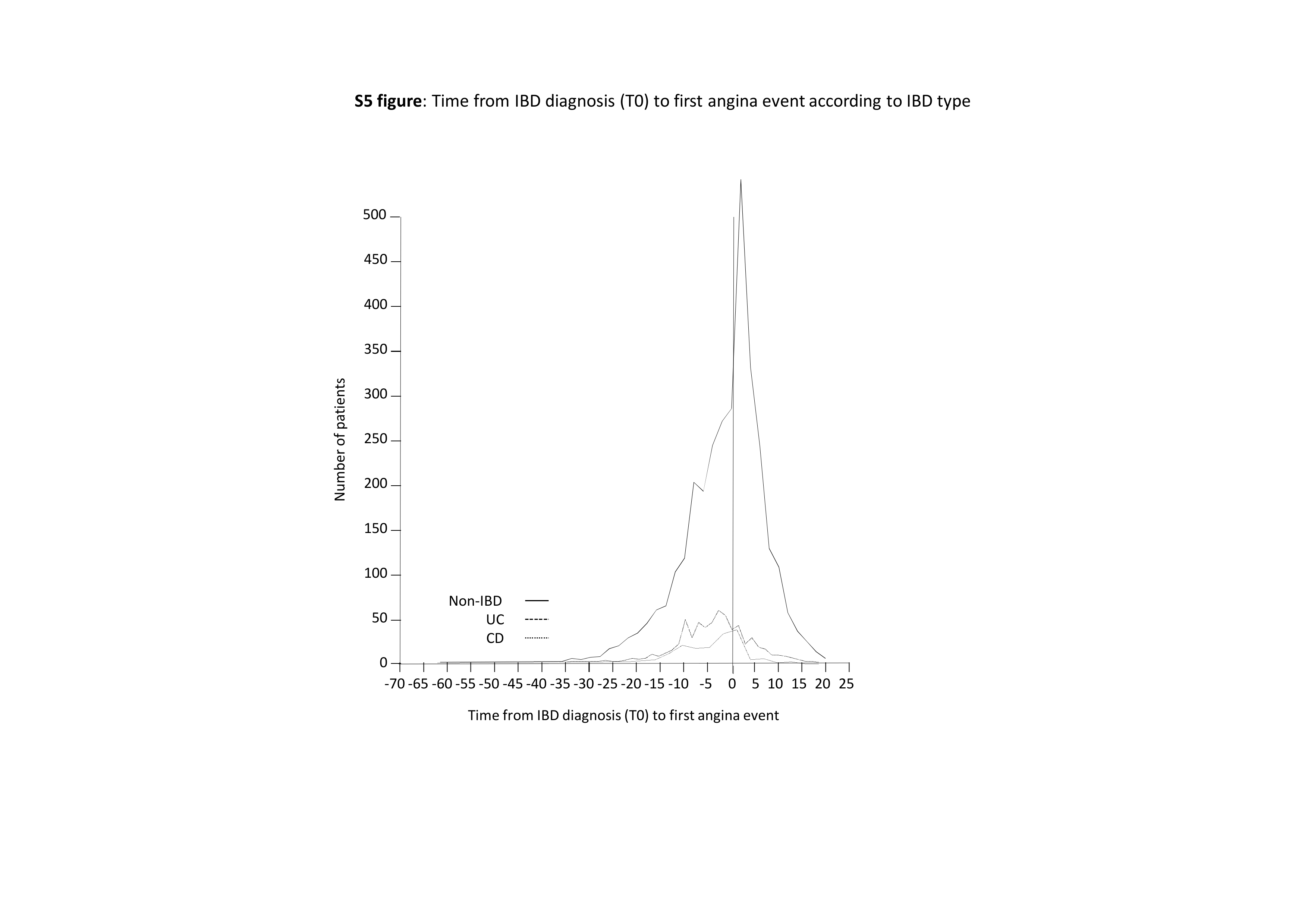

Supplement: S5 Fig — Total number of patients showing time from IBD diagnosis (T0) to the first Angina (sub-group of CVD) event according to IBD type. (TIF) [file pone.0139745.s005.tif]

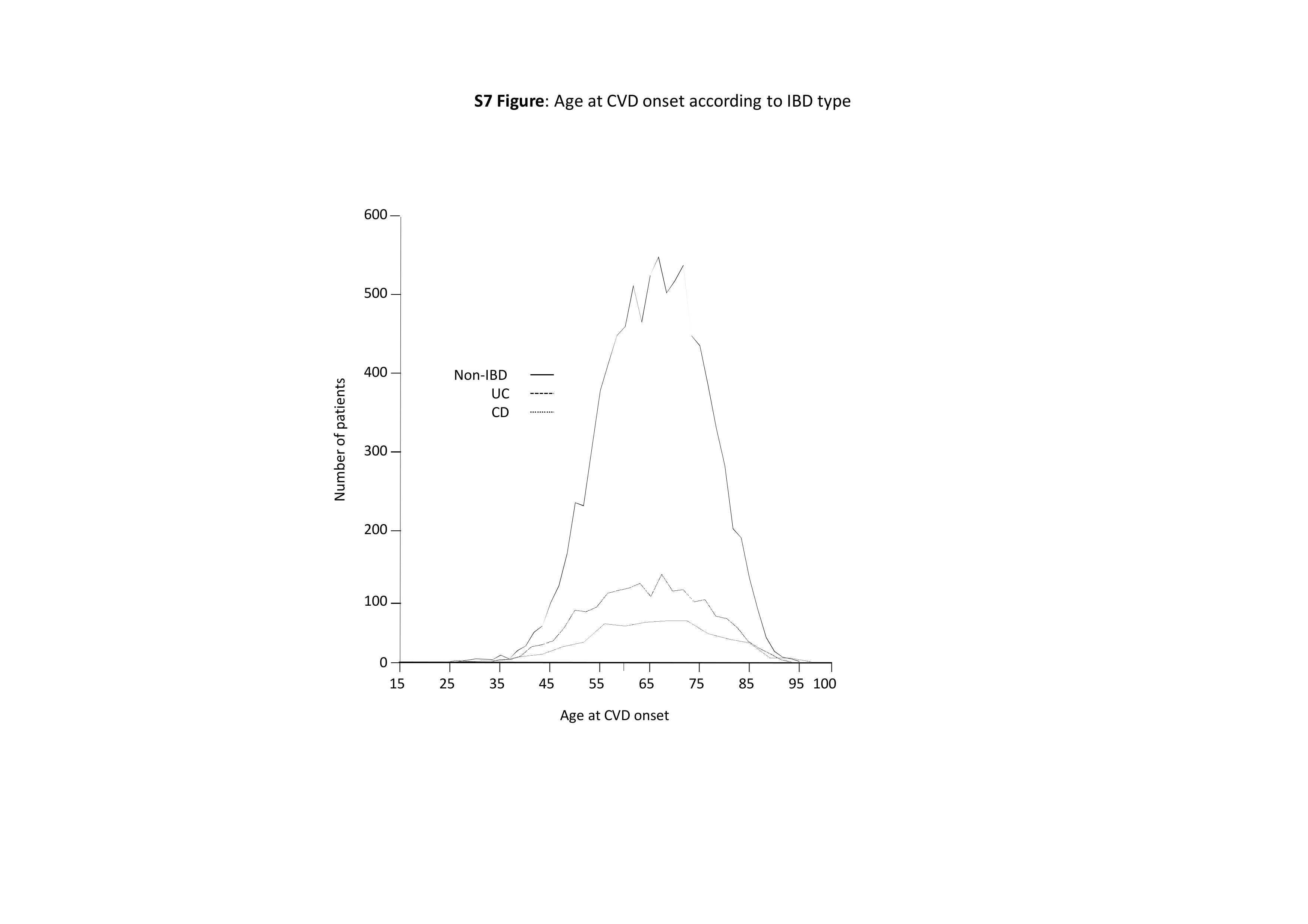

Supplement: S6 Fig — Total number of patients showing time from IBD diagnosis (T0) to the first Myocardial Infarction (sub-group of CVD) event according to IBD type. (TIF) [file pone.0139745.s006.tif]

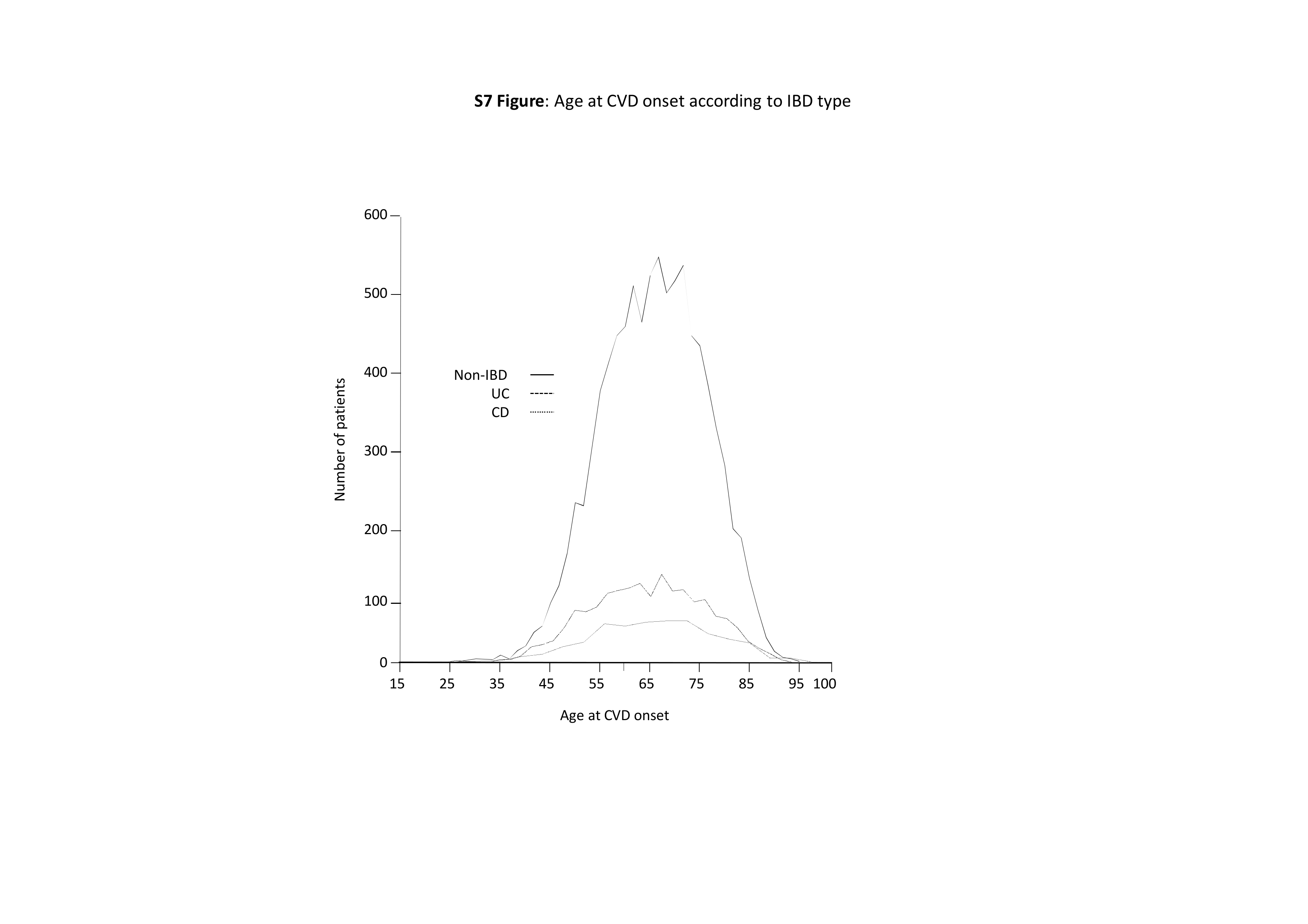

Supplement: S7 Fig — Total number of patients showing age at onset of Cardiovascular Disease (incorporating IHD, CHD, angina and MI) according to IBD type. (TIF) [file pone.0139745.s007.tif]
